# Cultural competence among nursing students and nurses working in acute care settings: a cross-sectional study

**DOI:** 10.1186/s12913-023-09103-5

**Published:** 2023-02-01

**Authors:** Selvedina Osmancevic, Franziska Großschädl, Christa Lohrmann

**Affiliations:** Medical University of Graz, Institute of Nursing Science, Universitätsplatz 4, 8010 Graz, Austria

**Keywords:** Cultural competence, Nurses, Nursing students, Influencing factors, Acute care, Cross-sectional study

## Abstract

**Background:**

The increasing cultural diversity in healthcare in European countries, including Austria, has highlighted the need to enhance nurses’ cultural competence. Assessing cultural competence and identifying relevant influencing factors can help to improve culturally competent care. The aim of this study was to assess the cultural competence of nurses and nursing students working in Austrian acute care settings and to identify influencing factors using the Cultural Competence Assessment scale.

**Methods:**

A cross-sectional design was used. Data collection was carried out in March 2021 with nurses and nursing students in the last year of their studies who were working in Austrian acute care settings. Descriptive analysis was applied to display the general characteristics of the study participants and the levels of their overall cultural competence. A multiple linear regression analysis was conducted to analyze the influencing factors of cultural competence.

**Results:**

The nurses’ cultural competence level was moderate to high (mean = 3.89; SD = .48). Their age, educational level, cultural diversity training and self-perceived cultural competence significantly influenced the level (F (6, 875) = 18.971, *p* < .0000, adj. R2 = 1.09).

**Conclusions:**

Providing culturally competent healthcare services for culturally diverse patients is essential for all healthcare professionals, and especially for nurses who spend the most time with patients. Effective interventions, such as educational training, need to be implemented in order to deliver culturally competent care and potentially reduce disparities in healthcare and improve patient outcomes.

**Supplementary Information:**

The online version contains supplementary material available at 10.1186/s12913-023-09103-5.

## Background

The increasing cultural diversity in healthcare in European countries, including Austria, has highlighted the need to enhance nurses’ cultural competence [1]. Cultural competence has been defined as “the dynamic process of acquiring the ability to provide effective, safe, and quality care to the patients through considering their different cultural aspects” [2]. Healthcare professionals need to understand and respect cultural differences and provide culturally competent care to demonstrate such competence [3]. Cultural competence has been identified as an important factor in reducing disparities in healthcare and improving patient outcomes (e.g., patient satisfaction) [4]. Culturally competent care is sensitive to the cultural implications of this care. It involves the meaningful, culture-based use of health and care knowledge to coordinate the needs of individuals or groups and helps them to acquire good health and well-being or to cope with illnesses, disorders, and death [5]. Studies have shown that nurses providing culturally competent nursing care have the potential to improve the quality of care [3, 4], to heighten patient satisfaction, and to challenge racism in healthcare [6], all of which leads to better health outcomes (e.g., treatment adherence) in patients from a diversity of cultural backgrounds [7]. Nurses with high levels of cultural competence can establish more effective communication with patients, which, in turn, can help in the development of appropriate treatments [8].

In 2021, 17.1% of the Austrian population was represented by people with a migrant background. The largest migrant group was Germans, followed by Romanians, Hungarians, and Croatians [9]. As the number of people with different cultural backgrounds in Austria has increased, the cultural diversity in the Austrian healthcare system has also increased. Despite the demonstrated importance of exhibiting cultural competence in terms of improving patient outcomes and nursing competencies, nurses working in the European countries of Germany, Czech Republic, Hungary, Slovakia, and Poland do not generally perceive themselves as culturally competent [10]. Most of the nurses (70.6%) indicated that they faced challenges while providing nursing care for patients with different cultural backgrounds. The main challenges mentioned were differences in language, religion, and a lack of cultural knowledge [10]. These findings show that assessments of and improvement in nurses’ cultural competence is necessary in Austria. Furthermore, by assessing and identifying various factors that influence cultural competence, researchers and healthcare professionals can design focus interventions (e.g., cultural diversity training programs) to meet the nurses’ needs [8]. The research on cultural competence in Austrian nurses, however, is still limited. Although some international studies have been carried out to investigate factors that influence cultural competence, these findings cannot be generalized to Austrian nurses due to a lack of evidence [8, 11]. In their study, Lin et al. (2021) found that cultural competence education acquired in nursing school has no influence on the cultural competence of registered nurses, but does seem to have an influence on pre-graduate nursing students [11]. Another study shows that nurses who are older and have received prior diversity training exhibit significantly higher levels of cultural competence [12]. These findings indicate that the assessment of Austrian nurses’ cultural competence and the identification of factors that influence this competence can help researchers and healthcare professionals to develop coherent interventions and to improve culturally competent care.

To our knowledge, no study has previously been carried out to assess the cultural competence of Austrian nurses with a psychometrically tested instrument. This study, therefore, was conducted to assess the cultural competence of nurses and nursing students working in Austrian acute care settings and to identify influencing factors.

## Methods

### Design

This cross-sectional study was conducted in March 2021 in Austrian acute care settings.

### Sampling method and data collection

Austrian nurses and nursing students in the last year of their studies who provided direct care for patients in acute care settings were invited to complete the questionnaire. We wanted to reach as many nurses as possible, and since the highest number of nurses work in acute care settings in Austria, we chose this setting for our research project. The researchers initially sent an invitation via e-mail to the directors of nursing staff in all Austrian acute care institutions and universities of applied sciences, explaining the purpose of the study. The recipients were politely requested to forward the e-mail invitation with the link to the online questionnaire to their nursing staff or nursing students. After two weeks, a reminder was sent out. Data collection was carried out online with LimeSurvey, a free and open source on-line statistical survey web application, in March 2021. We also shared information about the study via Facebook in order to reach as many nurses as possible. The survey enabled the application of the German version of the previously validated Cultural Competence Assessment scale (CCA-G) [13] and the collection of baseline demographics for these nurses and nursing students (age, gender, educational level). In addition, based on a survey of the literature [8, 12, 14, 15], we included specific questions to determine the number of years the nurses/students had worked, if they were multilingual, if they had taken part in a diversity training program,, if they had a migrant background, and their self-perceived cultural competence level. Persons of migration background were defined as persons whose parents were both born abroad. We distinguished further between first-generation and second-generation migrants. First-generation migrants are persons born abroad whose parents were also born abroad. Second-generation migrants are nationals born in the respective country whose parents were born abroad [16].

### Instrument

To assess the cultural competence level, we used the German version of the previously validated Cultural Competence Assessment scale (CCA-G) [13]. The self-reported CCA has been translated and been psychometrically tested in various languages (Italian, Spanish, Korean, and Slovakian). It has been found to be a reliable and valid instrument for the assessment of cultural competence in healthcare [12, 14, 17, 18]. The original CCA scale [19] was developed in the USA on the basis of the Cultural Competence Model developed by Schim and Miller. This scale consists of 25 items assigned to two subscales: cultural awareness and sensitivity (CAS) and culturally competent behavior (CCB). Cultural awareness refers to the professionals’ knowledge of differences and similarities between examples of cultural expression. Cultural competence behaviors refer to determining behaviors that are affected by diversity experiences made with culturally diverse people, cultural awareness, and sensitivity to the self and others [19]. The modified CCA-G consists of 14 items assigned to two subscales: cultural awareness and cultural competence behavior. The cultural awareness subscale consists of five items; these can be rated on 5-point Likert scale with answers ranging from totally agree to totally disagree. The cultural competence behavior subscale consists of nine items; these can be rated on a 5-point Likert scale with answers ranging from always to never. Higher scores indicate higher levels of cultural competence. The additional question about self-perceived cultural competence can be rated on a 5-point Likert scale, with answers ranging from very incompetent to very competent. Higher scores indicate higher levels of cultural competence.

### Data analysis

Data were analyzed using the statistical software program IBM SPSS Statistics 26 [20]. A descriptive analysis was performed to display the general characteristics of the study participants and the levels of their overall cultural competence with respect to the two subscales. Continuous variables were described as mean values and standard deviations, and categorical variables as frequencies and percentages. For the univariate analysis, Spearman’s rank correlation coefficient was used to measure the relationship between cultural competence and the continuous variables. The Mann–Whitney *U* test and Kruskal–Wallis test were performed to measure the relationship between cultural competence and categorical variables. The linearity of the continuous variable with respect to the logit of the dependent variable was assessed by applying the Box-Tidwell procedure, which adds an interaction term between the continuous independent variables and their natural logs to the regression equation. The independence of residuals was assessed by applying the Durbin-Watson statistic (range from 0 to 4; a value of approximately two indicates that no correlation exists between the residuals). Studentized residual values were used to detect outliers (values greater than three can be treated as outliers). Multicollinearity was recognized to occur if the variance inflation factors (VIF) were under four. After testing the assumptions for linear regression, a multiple linear regression analysis was conducted using an enter procedure to predict the dependent variable, i.e., the dependence of cultural competence on influencing factors identified by univariate analysis. Age, educational level, migrant background, multilingual ability, cultural diversity training experience, and self-perceived cultural competence were included as independent variables. Statistical significance was determined as *p* < 0.05.

## Results

Overall, 915 nurses and nursing students completed the questionnaire. Most of these were female (80.6%) with a mean age of 43 (± 10.93). Most of the nurses had already worked for more than ten years in nursing practice (70.8%). About one-third of the nurses described themselves as neither culturally competent nor incompetent (29.7%), and only a few nurses felt somewhat culturally incompetent (3.3%) or very incompetent (0.3%). Almost half of the nurses described themselves as culturally competent when providing nursing care (48.4%). An overview of the participants’ characteristics is given in Table 1.

**Table 1:** Cultural diversity training; Multilingualism; Migrant background need an indent- they need to be at the same level as mean age in years, female etc

Variable	Total sample *N* = 915
Mean age in years (SD)	43 (10.93)
Female (%)	80.6%
Education (%)	
• Nursing student (in an education program)	7.1%
• Nurse	59.9%
• Nurse with additional qualifications	33.0%
○ Bachelor’s degree	44.6%
○ Diploma/Master’s degree	55.1%
○ PhD	0.4%
Years of professional experience (%)	
• < 10 years	29.2%
• > 10 years	70.8%
Cultural diversity training (%)	48.5%
Multilingualism (%)	54.5%
Migrant background (%)	13.0%
• First generation	64.0%
• Second generation	36.0%
Self-perceived cultural competence (%)	
• Very incompetent	0.3%
• Somewhat incompetent	3.3%
• Neither competent nor incompetent	29.7%
• Somewhat competent	48.4%
• Very competent	18.3%

*SD* Standard deviation

Overall, the nurses’ level of cultural competence with regard to their CCA-G scores was moderate (mean = 3.89; SD = 0.48). The nurses´ responses showed that they had a high level of cultural awareness (4.42 ± 0.45). The highest-rated awareness item was “I believe that everyone, regardless of their cultural heritage, should be treated with respect” (4.92 ± 0.38). Responses collected regarding the cultural behavior subscale showed that the nurses exhibited a moderate level of culturally competent behaviors (3.59 ± 0.59). The highest-rated behavior item was “I gladly accept feedback from clients on how I relate to people from different cultures” (4.33 ± 0.85), and the lowest-rated was “I have access to textbooks and other materials that help me learn more about people from different cultures” (2.53 ± 1.27) (Table 2).

**Table 2:** Cultural Competence item means of the CCA-G

Cultural Competence items	Mean (SD)
**Cultural Awareness**	4.42 (.45)
Spiritual and religious beliefs are important aspects of many cultural groups	4.23 (.79)
Individuals can identify with more than one cultural group	4.05 (.95)
I believe that everyone, regardless of their cultural heritage, should be treated with respect	4.92 (.38)
I understand that people from different cultures can define the concept of “health care” in different ways	4.47 (.79)
I think that my knowledge about different cultural groups can help me in my work with individuals, families, and groups	4.44 (.78)
**Cultural Competence Behavior**	3.59 (.59)
I seek information about cultural needs when I meet new people at my work or educational institution	3.02 (1.08)
I have access to textbooks and other materials that help me learn more about people from different cultures	2.53 (1.27)
I ask people to tell me about their expectations regarding nursing care services	3.36 (1.16)
I avoid using generalizations to apply stereotypes to groups of people	4.14 (.78)
I recognize potential barriers to healthcare services that different people might encounter	3.64 (.69)
I remove barriers regarding nursing services affecting people from different cultural backgrounds, when I identify them	3.86 (.84)
I remove barriers for people from different cultures, when they tell me about them	3.84 (.84)
I gladly accept feedback from clients on how I relate to people from different cultures	4.33 (.85)
I find possibilities to adapt my nursing services to fit the cultural preferences of individuals and groups	3.60 (.85)

*SD* Standard deviation

### Factors influencing cultural competence

A multiple regression analysis was conducted to identify which of the factors age, educational level, migrant background, multilingual ability, cultural diversity training experience, and self-perceived cultural competence influenced the cultural competence of nurses. The assumptions of linearity, independence of errors, homoscedasticity, unusual points, and normality of residuals were met. The multiple regression analysis results show that four of these factors (age, educational level, cultural diversity training experience, and self-perceived cultural competence) were identified as factors that influenced cultural competence. These variables statistically significantly influenced cultural competence, F (6, 875) = 18.971, *p* < 0.0000, adj. R2 = 1.09, (Table 3). The regression analysis results show that older nurses who held an undergraduate degree or had completed higher education, participated in a diversity training program, and perceived themselves as somewhat or very culturally competent tended to have higher cultural competence levels.

**Table 3:** Influencing factors for cultural competence

Independent variables	B	Std. Error	Beta	*p*-value	VIF
(Constant)	48.924	2.331		.000	
Age	.048	.021	.078	.020	1.109
Educational level	.749	.373	.064	.045	1.011
Cultural diversity training experience	-2.000	.449	-.149	.000	1.109
Multilingual ability	-.671	.458	-.050	.144	1.148
Migrant background	-.849	.665	-.043	.202	1.108
Self-perceived cultural competence	1.940	.283	.225	.000	1.072

*B* Unstandardized regression coefficient, *SEB* Standard error of the coefficient, *Beta* Standardized coefficient, *CI* Cconfident interval, *p*-value significance level

## Discussion

Assessing nurses’ cultural competence is of primary importance in countries like Austria, where ethnic and cultural diversity is growing rapidly and, therefore, is being increasingly encountered by professionals working in the healthcare sector. This study provides the first insights into the cultural competence levels of Austrian nurses and nursing students working in acute care settings. Overall, the participants’ cultural competence level was moderate to high, and participants showed high levels of cultural awareness (mean = 4.42; SD = 0.45) as well as a moderate level of cultural competence behavior (mean = 3.59; SD = 0.59). Our results are comparable with results from studies performed in two neighboring states, Italy and Slovakia, where the authors used the same instrument to assess cultural competence in nurses [12, 21]. Nurses who exhibit high levels of cultural awareness generally have the ability to recognize differences and similarities of cultural expression, but also to display an open attitude toward and respect for cultural differences. These abilities are essential for achieving cultural competence in practice [19, 22]. The implementation of cultural assessment in the workplace, asking patients about their needs and expectations for care, adapting interventions to respect cultural practices, and seeking additional information and resources are all hallmarks of cultural competence [22]. The moderate level of cultural competence behavior among nurses identified in this study indicates that Austrian nurses are still restricted in their abilities to assess and address culturally needs in their daily practice.

In our study, the nurses’ self-perceived cultural competence levels were higher than those reported by nurses in Italy [21] and Slovakia [12]. While in Austria, 66.7% of the participants felt somewhat or very culturally competent, only 62% of Slovakian nurses [12] and 44% of Italian nurses [21] described themselves as somewhat or very culturally competent. However, when these levels are compared with those reported from countries like the United States, where cultural competence has been taught and encouraged in the nursing practice for decades, the self-perceived cultural competence in European countries still seems to be low. The study by Doorenbos et al. (2016) clearly demonstrates this, where 92.7% of the participants reported that they felt either somewhat competent or very competent [23].

### Factors influencing cultural competence

Multiple regression analysis results show that age, educational level, previous cultural diversity training experience, and self-perceived cultural competence significantly influenced the level of cultural competence among nurses. The data analysis results show that about a half of the nurses who had participated in cultural diversity training events or programs showed higher scores on the CCA-G. According to the Cultural Competence Model, our results suggest that the more training nurses receive, the more frequently they practice culturally competent behaviors. These findings agree with those from several other studies [8, 11, 19, 23] which found that prior diversity training influenced cultural competence levels, indicating that cultural competence training might help to support nurses when caring for patients with different cultural backgrounds. However, Cicolini et al. (2015) and Cerveni et al. (2020) found that prior diversity training did not influence cultural awareness; instead, their findings indicated that it influenced the cultural competence behavior subscale [12, 21]. Developing nurses’ cultural competence by offering specific education should be recognized as a priority because nurses work closely with people in increasingly diverse healthcare settings [24]. However, much critique has been expressed, particularly about educational training not being up to date and leading to stereotyping. Cultural competence training programs should be based on accepted educational theories and theories that explain the development of cultural competence. Furthermore, training programs should be developed to emphasize patient-centered care, to support the nurses’ ability to treat the patients as individuals, and to encourage them to reflect on their own cultural backgrounds [24]. Beside educational programs, organizational approaches need to be developed and applied to improve cultural competence, considering cost factors and aspects related to individual and organizational contexts [25]. Previous studies have shown that healthcare facilities must provide ongoing educational programs to ensure effective, culturally competent nursing care and to encourage nurses to continually increase their cultural competence [1, 24]. Healthcare organizations should act to address challenges associated with cultural diversity and to ensure that nursing staff are provided with high-quality continuing education offers in cultural competence [24]. This means that improvements in both the nurses’ cultural competence levels and organizational factors are needed to provide equally high-quality care for all patients.

The results show that the educational level rather than length of working experience influenced cultural competence, with a higher educational level indicating higher levels of cultural competence. Our study findings support the findings of other studies [8, 11, 12] which show that increasing cultural competence depends on the nurses´ education level. This finding signifies that increasing cultural competence does not depend on previous diversity trainings but also on the nurses’ educational level. According to Cicolini et al. (2015), this influence might be due to the greater exposure to cultural diversity that occurs in higher education [21].

The study findings also show that nurses who perceived themselves as very or somewhat competent had higher cultural competence levels than nurses who perceived themselves as rather incompetent or neutral. These results agree with those of other studies which show that nurses who rated themselves as having higher levels of self-perceived cultural competence had higher levels of cultural competence ^12,21,23^. Increasing an awareness of one’s own cultural properties can be useful in cross-cultural encounters in a healthcare setting and can improve the cultural competence of nurses.

The strength of our study is its large sample size and inclusion of nurses and nursing students from different parts of Austria. The study participants were included using a convenience sampling, however, which limits the overall generalizability of the findings. The cross-sectional design presents a further limitation. Studies with a longitudinal design should be undertaken to increase our understanding of the cultural competence of nurses in all Austria healthcare settings. Another limitation of this study is including nursing students, which may have influenced our results to a certain degree, even if they constitute only 7% of our sample. However, we invited only last-semester nursing students who already have substantial experience in nursing care. Some of the influencing factors, like years of work experience or multilingualism, could have been presented in more detail. Creating a finer subdivision regarding years of experience or assessing which other language(s) the participants can speak might have provided a more detailed insight on the results, and, maybe, might have even yielded slightly differing study results.

## Conclusions

The need to provide culturally competent healthcare in Europe and specifically in Austria is changing due to ongoing demographic changes. Results of this study show that the nurses’ cultural competence level was moderate to high. Older nurses who held an undergraduate degree or had completed higher education, participated in a diversity training program, and perceived themselves as somewhat or very culturally competent tended to have higher cultural competence levels. Effective interventions, such as educational training events or programs designed to meet nurses’ needs, need to be implemented in Austrian acute care settings in order to promote culturally competent nursing care. Further research is deemed necessary to examine how other relevant variables influence cultural competence.

## Supplementary Information

**Additional file 1.** The German Version of the Cultural Competence Assessment (CCA-G).

## Data Availability

The datasets generated and/or analyzed during the current study are not publicly available because in the study protocol no data sharing was stated, but are available from the corresponding author on reasonable request.

## Abbreviations

CCA-G: Cultural Competence Assessment- German Version
CCA: Cultural Competence Assessment
CAS: Cultural Awareness Scale
CCB: Cultural Competence Behaviour
VIF: Variance Inflation Factors
B: Unstandardized regression coefficient
SEB: Standard error of the coefficient
Beta: Standardized coefficient
CI: Confidence Interval

## References

[1] Jongen C, McCalman J, Bainbridge R (2018). Health workforce cultural competency interventions: a systematic scoping review. BMC Health Serv Res.

[2] Sharifi N, Adib-Hajbagheryb M, Najafic M. Cultural competence in nursing: A concept analysis. International Journal of Nursing Studies. 2019;99doi:10.1016/j.ijnurstu.2019.103386 10.1016/j.ijnurstu.2019.103386 31404821

[3] Cai D-Y (2016). A concept analysis of cultural competence. Int J Nurs Sci.

[4] Kaihlanen AM, Hietapakka L, Heponiemi T (2019). Increasing cultural awareness: qualitative study of nurses' perceptions about cultural competence training. BMC Nurs.

[5] McFarland M, Wehbe-Alamah H (2018). Transcultural Nursing Concepts.

[6] Antón-Solanas I, Huércanos-Esparza I, Hamam-Alcober N (2021). Nursing Lecturers’ Perception and Experience of Teaching Cultural Competence: A European Qualitative Study. Int J Environ Res Public Health.

[7] Ervin N, Bickes J, Schim SM (2006). Environments of care: a curriculum model for preparing a new generation of nurses. J Nurs Educ.

[8] Cai D, He W, Klug D (2021). Cultural competence among nurses and its influencing factors: A cross-sectional study. Nurs Health Sci.

[9] Statistik-Austria. Österreich. Zahlen. Daten. Fakten. 2022. https://www.statistik.at/web_de/services/oesterreich_zahlen_daten_fakten/index.html

[10] Červený M, Siaki LA, McGee P, Kilíková M (2019). Perception of European nurses of culturally-appropriate health care – a cross-sectional study. Med Og Nauk Zdr.

[11] Lin HL, Guo JL, Chen HJ, Liao LL, Chang LC (2021). Cultural competence among pre-graduate nursing students, new graduate nurses, nurse mentors, and registered nurses: A comparative descriptive study. Nurse Educ Today.

[12] Cerveny M, Dimunova L, Della Pelle C (2020). Self-Reported Cultural Competence of Nurses Providing Nursing Care in Slovakia. J Nurs Scholarsh.

[13] Osmancevic S, Großschädl F, Stijic M, Lohrmann C (2022). The German version of the Cultural Competence Assessment (CCA-G): cross-cultural adaptation and validation study in Austrian acute care settings. BMC Nurs.

[14] Caricati L, Dicembrino RB, Gionti L, Petre L, Ungurean L (2015). Cultural Competence Assessment Instrument: Initial Italian validation and proposed refinement. Acta Biomed.

[15] Shen Z (2015). Cultural competence models and cultural competence assessment instruments in nursing: a literature review. J Transcult Nurs.

[16] Nations U. Recommendations for the 2020 Censuses of Population and Housing. presented at: Conference of European Statisticians 2015. New York and Geneva: UNECE (United Nations Economic Commission for Europe).

[17] Chae D, Kang KH, Benkert R, Doorenbos AZ (2018). Evaluation of the psychometric properties of the Korean version of the Cultural Competence Assessment. Jpn J Nurs Sci.

[18] Raigal-Aran L, Ferré-Grau C, Belzunegui-Eraso A (2019). The Spanish version of the Cultural Competence Assessment (CCA-S): Transcultural validation study and proposed refinement. Nurse Educ Today.

[19] Schim SM, Doorenbos AZ, Miller J, Benkert R (2003). Development of a Cultural Competence Assessment instrument. J Nurs Meas Spring-Summer.

[20] IBM Corp. IBM SPSS Statistics for Windows, version 26.0. Armonk: IBM Corp; 2019.

[21] Cicolini G, Della Pelle C, Comparcini D (2015). Cultural Competence Among Italian Nurses: A Multicentric Survey. J Nurs Scholarsh.

[22] Doorenbos AZ, Schim SM, Benkert R, Borse NN (2005). Psychometric evaluation of the cultural competence assessment instrument among healthcare providers. Nurs Res.

[23] Doorenbos AZ, Morris AM, Haozous EA (2016). Assessing Cultural Competence Among Oncology Surgeons. J Oncol Pract.

[24] Oikarainen A, Mikkonen K, Kenny A (2019). Educational interventions designed to develop nurses’ cultural competence: A systematic review. Int J Nurs Stud.

[25] Truong M, Paradies Y, Priest N (2014). Interventions to improve cultural competency in healthcare: a systematic review of reviews. BMC Health Serv Res.

