# Supplementary material for: Cultural competence among nursing students and nurses working in acute care settings: a cross-sectional study
Source: BMC Health Serv Res. 2023 Feb 1;23:105. doi: 10.1186/s12913-023-09103-5 (PMC9890795; doi:10.1186/s12913-023-09103-5)
Supplement: Supplementary file 1 — Additional file 1. The German Version of the Cultural Competence Assessment (CCA-G). [file 12913_2023_9103_MOESM1_ESM.docx]

Appendix 1: The German Version of the Cultural Competence Assessment (CCA-G)

| **Cultural Awareness** |
| --- |
| Spiritual and religious beliefs are important aspects of many cultural groups. |
| Individuals can identify with more than one cultural group. |
| I believe that everyone, regardless of their cultural heritage, should be treated with respect. |
| I understand that people from different cultures can define the concept of “health care” in different ways. |
| I think that my knowledge about different cultural groups can help me in my work with individuals, families, and groups. |
| **Cultural Competence Behavior** |
| I seek information about cultural needs when I meet new people at my work or educational institution. |
| I have access to textbooks and other materials that help me learn more about people from different cultures. |
| I ask people to tell me about their expectations regarding nursing care services. |
| I avoid using generalizations to apply stereotypes to groups of people. |
| I recognize potential barriers to healthcare services that different people might encounter. |
| I remove barriers regarding nursing services affecting people from different cultural backgrounds, when I identify them. |
| I remove barriers for people from different cultures, when they tell me about them. |
| I gladly accept feedback from clients on how I relate to people from different cultures. |
| I find possibilities to adapt my nursing services to fit the cultural preferences of individuals and groups. |
